# The potential of targeting microRNA-23b-3p signaling in Alzheimer’s disease: opinions on recent findings

**DOI:** 10.3389/fphar.2023.1245352

**Published:** 2023-07-12

**Authors:** Chenyu Wang, Zhongdi Cai, Zhuorong Li, Rui Liu

**Affiliations:** Institute of Medicinal Biotechnology, Chinese Academy of Medical Sciences and Peking Union Medical College, Beijing, China

**Keywords:** Alzheimer’s disease, microRNA-23b-3p, microRNA therapy, glycogen synthase kinase-3β, tau protein

## 1 Introduction

Alzheimer’s disease (AD) is a predominant form of dementia characterized by progressive cognitive impairments including behavioral changes, language disorder, and mental symptoms, ultimately leading to death (Wu et al., 2022). Since first described in 1907 by Alois Alzheimer, awareness of the pathological characteristics and clinical manifestations of AD has improved. However, the etiology of AD remains uncertain and is considered the result of interactions among biological, genetic, and environmental factors. A wide range of pathological hypotheses have been proposed, mainly centered on the deposition of amyloid-β (Aβ), neurofibrillary tangles induced by hyperphosphorylated tau, synaptic dysfunction, neuroinflammation, and neurotransmitter imbalance (Decourt et al., 2022). However, at present, only symptomatic treatments exist for AD.

MicroRNAs (miRNAs) are a type of regulatory non-coding RNAs (ncRNAs) important in modulating gene expression (Ren et al., 2022). miRNAs are 19–24 nt length single-stranded RNAs with a role in post-transcriptional gene silencing. Over 38,000 miRNAs have been identified to date (https://www.mirbase.org/), approximately 68 miRNAs of which have been comprehensively studied and associated with the onset and development of AD (Supplementary Table S1). Among these, miR-23b-3p has been broadly investigated in peripheral circulatory systems and various AD models. In this opinion article, informed by published data and our own findings, we outline the role of miR-23b-3p in AD pathology and its biomarker potential and target properties for future miRNA-based applications in AD.

## 2 MiR-23b-3p plays a key role in AD

MiR-23b-3p is a member of the miR-23b family. Initially described in 2012, miR-23b-3p plays important roles in carcinogenesis (Grossi et al., 2018) and non-cancerous diseases, especially nervous system diseases like ischemic stroke (Wu et al., 2017), seizure (Zhan et al., 2016), Parkinson’s disease (Geng et al., 2023), peripheral nerve regeneration (Xia et al., 2020), and neuroinflammation-related diseases (Burrows et al., 2023). The first reported relationship between miR-23b-3p and AD was altered expression of miR-23b-3p in AD patients (Lugli et al., 2015). The role and underlying mechanism of miR-23b-3p, as well its potential applications in AD, have also been investigated by our group.

Our recent research indicates that miR-23b-3p plays a neuroprotective role in AD. Low expression of miR-23b-3p was revealed to correlate with AD progression (Liu et al., 2021). Downregulation of miR-23b-3p has been reported with disease development in various AD models, including in the hippocampus and cortex of amyloid precursor protein (APP)/presenilin 1 (PS1) mice and senescence-accelerated mouse prone 8 (SAMP8) mice at different ages (Liu et al., 2020). Decrease of miR-23b-3p in neuronal cells with Aβ toxicity also has a time-effect relationship (Jiang et al., 2022). Furthermore, the correlation analysis of plasma miR-23b-3p levels and mini-mental state examination scores in AD patients has a good clinical diagnostic value with 81.8% sensitivity and 92.9% specificity (Jiang et al., 2022).

Importantly, functional evidence of the beneficial roles of miR-23b-3p suggests that modifying its expression could counteract AD pathology. Intracerebral ventricular infusion of adeno-associated virus (AAV) constructs expressing miR-23b-3p in APP/PS1 mice rescued learning and memory deficits in the Morris water maze test (Jiang et al., 2020). The neurotic plaques forming Aβ and neurofibrillary composed of hyperphosphorylated tau are indicators of AD. It has been found that restoring miR-23b-3p levels via direct delivery of AAV-loaded miR-23b-3p into the brain or miR-23b-3p synthetic mimics transfected into neuronal cells both suppressed Aβ_1-42_ levels and tau phosphorylation against AD-related deficits, thereby inhibiting cell apoptosis (Jiang et al., 2022; Liu et al., 2022). In contrast, use of a miR-23b-3p inhibitor led to the opposite effects *in vitro*. These findings are in line with related reports for other neurological diseases that miR-23b alleviates hypoxia-induced neuronal apoptosis (Chen et al., 2014) and that upregulation of miR-23b-3p has positive effects on neuronal activity, plasticity, and cognitive functions like long-term potentiation and memory in response to music-listening (Nair et al., 2021). Together, these findings support that increasing miR-23b-3p level in the brain of AD models is potentially therapeutic, as it could concomitantly modify various aspects of AD’s hallmark pathology and potentially lead to amelioration of cognitive deficits.

## 3 MiR-23b-3p participates in multi-pathway crosstalk for AD therapy

**FIGURE 1:**
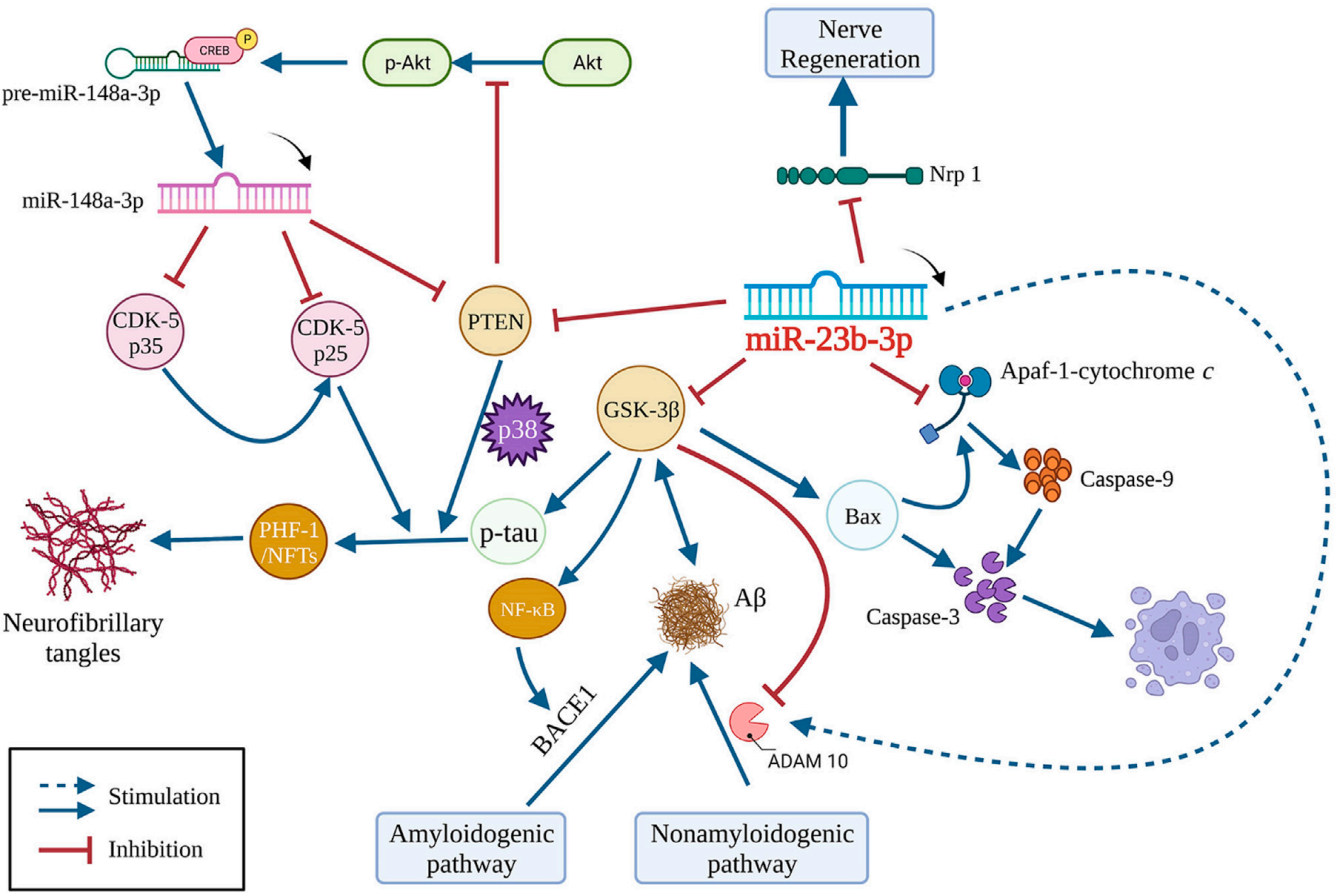
Multi-Pathway Crosstalk of miR-23b-3p in AD. Bax, Bcl-2-associated X protein; GSK-3β, glycogen synthase kinase-3β; Aβ, amyloid-β; BACE1, beta-site APP cleaving enzyme 1; ADAM10, disintegrin and metalloprotease 10; PTEN, phosphatase and tensin homolog; CDK-5, cyclin-dependent kinase-5; PHF-1, paired helical filament-1; NFTs, non-fungible tokens; Nrp 1, neuropilin 1; Apaf-1, apoptotic protease activating factor-1; CREB, cyclic adenosine monophosphate response element-binding protein; NF-κB, nuclear factor-κB.

Our findings suggest that miR-23b-3p is downregulated in AD, causing a loss of the effective inhibitory effect on the expression of its target gene glycogen synthase kinase 3 beta (GSK-3β). Activation of GSK-3β/tau hyperphosphorylation signaling is crucial in causing cognitive impairment (Lauretti et al., 2020). Increased GSK-3β activity is known to contribute to Aβ production by inducing the beta-site APP cleaving enzyme 1-mediated amyloidogenic pathway and downregulating disintegrin and metalloprotease 10 (ADAM10) in the nonamyloidogenic pathway (Liu et al., 2018; Ly et al., 2013). miR-23b-3p has been shown to directly suppress tau hyperphosphorylation by targeting GSK-3β and to indirectly reduce Aβ_1-42_ production by increasing the ADAM10-mediated nonamyloidogenic pathway that GSK-3β is involved in. GSK-3β is intimately associated with apoptosis. In line with this relationship, the GSK-3β/Bax/caspase-3 axis was shown to be inhibited by administering miR-23b-3p to both *in vitro* and *in vivo* AD models (Jiang et al., 2022). These findings suggest that miR-23b-3p multimodally inhibits Aβ generation, tau phosphorylation, and neuronal apoptosis by regulating one target, namely, GSK-3β, acting upstream of several signaling pathways (Figure 1).

Emerging evidence suggests additional targets for miR-23b-3p in non-cancerous diseases, such as neuropilin 1 (Xia et al., 2020), apoptotic protease activating factor-1 (Apaf-1) (Chen et al., 2014), α-synuclein (Geng et al., 2023), and phosphatase and tensin homolog (PTEN) (Guo et al., 2022). Among these, Apaf-1 mediates apoptosis via recruitment and activation of caspase-9 by binding with cytochrome *c*. Notably, PTEN is also a regulator of tau phosphorylation. As a negative regulator of PI3 kinase signaling, downregulation of PTEN-mediated Akt pathway induces activation of p38 MAPK, leading to tau phosphorylation (Zeng et al., 2021). The effect of PTEN on tau phosphorylation in AD is independent of GSK-3β and the tau phosphatases PP1 and PP2A (Kerr et al., 2006). PTEN also plays a role in a negative feedforward loop involving the miR-148a-3p-associated Akt/CREB pathway to recover cognitive impairment in AD (Zeng et al., 2021). In summary, various direct or indirect targets involved in AD pathogenesis have been shown to be regulated by miR-23b-3p, supporting the putatively central role of miR-23b-3p in AD.

## 4 Transformational potential of MiR-23b-3p from bench to bedside

Using artificial miR-23b-3p duplexes similar to its specific miRNA precursors, it is possible to affect a number of molecules in response to miR-23b-3p-mediated reduction of AD hallmarks, such as tau phosphorylation and Aβ production. GSK-3β is thought to be a key player in AD since its dysregulation is associated with several significant hallmarks of the disease, including tau hyperphosphorylation, Aβ accumulation, synaptic dysfunction, and microglia-mediated neuroinflammation (Lauretti et al., 2020). GSK-3β thus represents a promising disease-modifying target against AD, and several inhibitors are currently being used in pre-clinical and clinical studies. miR-23b-3p inhibits GSK-3β overexpression by specifically binding to *GSK3β* mRNA, upregulates inhibitory GSK-3β phosphorylation at the Ser9 site, and decreases GSK-3β overactivity in the brain (Jiang et al., 2022). A negative correlation has been observed between GSK-3β and miR-23b-3p in the cortex and hippocampus during AD progression, suggesting miR-23b-3p as a promising drug target. Specifically, we have started developing anti-AD candidate drugs to inhibit the GSK-3β/p-tau/Bax/caspase-3 pathways dependent on interference with miR-23b-3p (Li et al., 2023). However, clinical trials have not identified an effective and safe inhibitor of GSK-3β for AD due to its ubiquitous expression and multiple regulatory functions. Precise brain delivery of miR-23b-3p and specific regulation of GSK-3β may provide new prospects for the treatment of AD.

## 5 Final considerations

In this opinion article, we discuss the role and potential of targeting miR-23b-3p signaling in AD. To our knowledge, our research group was the first to report and thoroughly investigate the role and underlying mechanisms of miR-23b-3p in AD. Collectively, our studies support the cognitive enhancement effects of miR-23b-3p, identify the miR-23b-3p-elicited GSK-3β/p-tau/Bax/caspase-3 pathway as a promising network-based target, and investigate novel compounds based on miR-23b-3p interference for AD. Although specific miRNAs have potential as clinical biomarkers and drug targets, much work is still needed before miRNA-based applications are available. The combination of increased t-tau and p-tau levels and decreased Aβ levels in blood or cerebrospinal fluid is a sensitive and specific measure for AD diagnosis and monitoring. Before miR-23b-3p-associated candidate biomarkers can achieve routine clinical applications, they require critical evaluation, including clinical analysis, validity, and utility. miRNA-based therapeutics are currently in the development pipeline, along with other oligonucleotide-based approaches. However, there are still many future challenges for characterizing miR-23b-3p-mediated optimal target pathways and systematically mapping on- and off-target toxic effects. Despite these issues, we will continue to pursue this line of research and strive to incorporate interventions targeting miR-23b-3p into clinical diagnosis and treatment of AD.
