# Supplementary material for: The potential of targeting microRNA-23b-3p signaling in Alzheimer’s disease: opinions on recent findings
Source: Front Pharmacol. 2023 Jul 12;14:1245352. doi: 10.3389/fphar.2023.1245352 (PMC10368870; doi:10.3389/fphar.2023.1245352)
Supplement: Supplementary file 1 [file DataSheet1.PDF]

Supplementary Material

The Potential of Targeting MicroRNA-23b-3p Signaling in Alzheimer's Disease: Opinions on Recent Findings

Chenyu Wang[1†], Zhongdi Cai[1†], Zhuorong Li[1], Rui Liu[*1]

[†] These authors contributed equally to this work.

[*] **Correspondence:**
Rui Liu
liurui@imb.pumc.edu.cn, +86 10 67087731

1 Supplementary Table

Table S1. A brief summary of miRNA research in AD

MicroRNA	Expression Changes in AD	AD Pathologies	References
miR-10b-5p	Upregulation	neuronal apoptosis, inflammation<br><br>(*in vivo*)	Ruan, Z., et al. (2021). J Drug Target. 29 (5):531-540.
miR-16	Upregulation	Aβ production and deposition, tau phosphorylation, inflammation, neuronal cell apoptosis<br><br>(*in vitro* and *in vivo*)	Parsi, S., et al. (2015). Mol Ther Nucleic Acids. 4 (10):e256.<br><br>Kim, Y.J., et al. (2020). Exp Gerontol. 136:110954.
miR-22	Downregulation	neuroinflammation, apoptosis, Aβ deposition<br><br>(*in vitro* and *in vivo*)	Han, C., et al. (2020). Brain Behav. 10 (6):e01627. doi: 10.1002/brb3.1627.<br><br>Zhai, L., et al. (2021). J Cell Mol Med. 25 (15):7513-7523.

miR-23b	Downregulation	Aβ production, tau phosphorylation<br>(*in vitro* and *in vivo*)	Jiang, H., et al. (2022). Mol Ther Nucleic Acids. 28:539-557.<br>Pan, K., et al. (2021). Neuropharmacology. 196:108671.
miR-25	Upregulation	apoptosis<br>(*in vitro* and *in vivo*)	Duan, Q., et al. (2019). J Cell Biochem. 120 (9):15891-15905.
miR-26a-5p	Downregulation	tau phosphorylation, Aβ accumulation<br>(*in vivo*)	Liu, Y., et al. (2020). Curr Neurovasc Res. 17 (3):241-248.
miR-27a	Upregulation	neuronal apoptosis, inflammation<br>(*in vitro* and *in vivo*)	Hu, Y., et al. (2021). Hum Exp Toxicol. 40 (12_suppl):S347-s358.
miR-29	Downregulation	Aβ generation<br>(*in vitro* and *in vivo*)	Hébert, S.S. et al. (2008). Proc Natl Acad Sci U S A. 105 (17):6415-6420.
miR-30a-5p	Upregulation	neuronal injury and apoptosis, Aβ overproduction and accumulation<br>(*in vitro* and *in vivo*)	Sun, T., et al. (2022). Pharmacol Res. 178:106153.
miR-31	Downregulation	Aβ deposition<br>(*in vivo*)	Barros-Viegas, A.T., et al. (2020). Mol Ther Nucleic Acids. 19:1219-1236.
miR-33	Upregulation	Aβ secretion	Kim, J., et al. (2015). J Neurosci. 35 (44):14717-

		(*in vitro* and *in vivo*)	14726.
miR-34a	Downregulation	Aβ accumulation, tau phosphorylation, apoptosis<br><br>(*in vitro* and *in vivo*)	Li, P., et al. (2020). J Neurol Sci. 413:116793.<br><br>Sarkar, S., et al. (2019). Brain Res. 1721:146327.
miR-96-5p	Upregulation	Aβ production<br><br>(*in vitro* and *in vivo*)	Zhu, M., et al. (2021). J Alzheimers Dis. 83 (1):367-377.
miR-98	Upregulation	Aβ formation, tau phosphorylation, neuroinflammation<br><br>(*in vitro* and *in vivo*)	Hu, Y.K., et al. (2013). Neurosci Bull. 29 (6):745-751.<br><br>Song, C., et al. (2021). Faseb j. 35 (6):e21658.
miR-99b-5p	Downregulation (early stages)<br><br>Upregulation (late stages)	cell apoptosis<br><br>(*in vitro* and *in vivo*)	Ye, X., et al. (2015). Front Aging Neurosci. 7:210.
miR-100-5p	Downregulation (early stages)<br><br>Upregulation (late stages)	cell apoptosis<br><br>(*in vitro* and *in vivo*)	Ye, X., et al. (2015). Front Aging Neurosci. 7:210.
miR-101	Downregulation	tau phosphorylation, dendritic abnormalities, Aβ accumulation<br><br>(*in vitro* and *in vivo*)	Liu, D., et al. (2017). Mol Ther. 25 (3):752-764.<br><br>Vilardo, E., et al. (2010). J Biol Chem. 285 (24):18344-18351.
miR-106b	Downregulation	tau phosphorylation<br><br>(*in vitro* and *in vivo*)	Liu, W., et al (2016). Biochem Biophys Res Commun. 478 (2):852-857.
miR-107	Downregulation	Aβ accumulation, tau phosphorylation, cell apoptosis	Jiao, Y., et al. (2016). Neuropharmacology. 108:332-344.

		(*in vitro* and *in vivo*)	Shu, B., Zhang, X., Du, G., Fu, Q., Huang, L. (2018). Int J Mol Med. 41 (3):1665-1672.
miR-124	Upregulation	tau phosphorylation, cell apoptosis, Aβ deposition, inflammation<br><br>(*in vitro* and *in vivo*)	Garcia, G., et al. (2022). Front Pharmacol. 13:833066.<br><br>Hou, T.Y., et al. (2020). J Neurochem. 154 (4):441-457.<br><br>Zhou, Y., et al. (2019). J Alzheimers Dis. 67 (2):571-581.
miR-125b	Upregulation	tau phosphorylation, neuron apoptosis, neuroinflammation<br><br>(*in vitro* and *in vivo*)	Banzhaf-Strathmann, J., et al. (2014). Embo j. 33 (15):1667-1680.<br><br>Li, P., et al. (2020). J Neurol Sci. 413:116793.<br><br>Ma, P., et al. (2019). Curr Alzheimer Res. 16 (7):596-612.
miR-128	Upregulation	Aβ accumulation and generation, tau phosphorylation, neuroinflammation<br><br>(*in vitro* and *in vivo*)	Li, S., et al. (2023). CNS Neurosci Ther.<br><br>Liu, Y., et al. (2019). Eur J Pharmacol. 843:134-144.
miR-129	Downregulation	neuron apoptosis, neuroinflammation<br><br>(*in vivo*)	Li, Z., et al. (2020). Dement Geriatr Cogn Disord. 49 (2):163-169.<br><br>Sun, W., et al. (2020). Mol Neurobiol. 57 (12):5044-5055.
miR-132	Upregulation	neurons cell apoptosis, tau phosphorylation, Aβ production and	Liu, D.Y., et al. (2019). Eur Rev Med Pharmacol Sci. 23 (19):8523-8532.<br><br>Salta, E., et al. (2016).

		accumulation<br>(*in vitro* and *in vivo*)	EMBO Mol Med. 8 (9):1005-1018.
miR-134	Upregulation	neuroinflammation<br>(*in vivo*)	Abozaid, O.A.R., et al. (2022). Biol Trace Elem Res. 200 (12):5104-5114.
miR-135a	Downregulation	synaptic disorder, neuroinflammation, neuron apoptosis<br>(*in vivo*)	Chu, Y.Y., et al. (2016). Mol Neurobiol. 53 (6):4173-4188.<br>Zheng, K., et al. (2021). Nat Commun. 12 (1):1903.
miR-137	Downregulation	tau hyperphosphorylation<br>(*in vitro* and *in vivo*)	Jiang, Y., et al. (2018). Med Sci Monit. 24:5635-5644.
miR-138	Upregulation	Aβ production, tau phosphorylation, inflammation, synaptic dysfunction<br>(*in vitro* and *in vivo*)	Boscher, E., et al. (2019). J Alzheimers Dis. 68 (3):1243-1255.<br>Boscher, E., et al. (2020). Front Neurosci. 14:591138.
miR-139	Upregulation	neuroinflammation<br>(*in vitro* and *in vivo*)	Tang, Y., et al. (2017). Genet Mol Res. 16 (1). doi: 10.4238/gmr16019166.
miR-140	Upregulation	Aβ production, tau phosphorylation, neuroinflammation<br>(*in vitro* and *in vivo*)	Khodabakhsh, P., et al. (2023). CNS Neurosci Ther. 29 (1):91-103.<br>Liang, C., et al. (2021). Neurosci Lett. 758:135674.

miR-142-5p	Upregulation	Aβ formation,tau phosphorylation, neuroinflammation, neurons apoptosis<br><br>(*in vivo*)	Liang, W., et al. (2023). Brain Res Bull. 192:107-114.
miR-143-3p	Downregulation	tau phosphorylation, Aβ production<br><br>(*in vitro* and *in vivo*)	Wang, L., et al. (2022). Int J Mol Sci. 23 (14).
miR-146a	Upregulation	tau phosphorylation, neuroinflammation, glia activation, Aβ deposition<br><br>(*in vitro* and *in vivo*)	Mai, H., et al. (2019). Mol Ther Nucleic Acids. 18:681-695.<br><br>Wang, G., et al. (2016). Sci Rep. 6:26697.
miR-148a-3p	Downregulation	tau phosphorylation<br><br>(*in vitro* and *in vivo*)	Zeng, L., et al. (2021). Mol Ther Nucleic Acids. 27:256-275.
miR-155	Upregulation	Aβ deposition, neuron regeneration, neuroinflammation<br><br>(*in vitro* and *in vivo*)	Guedes, J.R., et al. (2014). Hum Mol Genet. 23 (23):6286-6301.<br><br>Wang, W., et al. (2022). J Neuropathol Exp Neurol. 81 (1):16-26.
miR-181	Downregulation	tau hyperphosphorylation, neuronal pyroptosis, neuroinflammation, Aβ accumulation<br><br>(*in vitro* and *in vivo*)	Wang, T., et al. (2023). Behav Brain Res. 447:114387.<br><br>Wu, Q., et al. (2019). Aging (Albany NY). 11 (16):6120-6133.<br><br>Yan, Y., et al. (2020). J Gene

			Med. 22 (12):e3268.
miR-188-3p	Downregulation	neuroinflammation, Aβ production<br><br>(*in vivo*)	Zhang, J., et al. (2014). J Neurosci. 34 (45):14919-14933.
miR-191-5p	Downregulation	tau phosphorylation, Aβ generation, and neuronal cell death<br><br>(*in vitro* and *in vivo*)	Wang, L., et al. (2022). ACS Chem Neurosci. 13 (24):3554-3566.
miR-195	Downregulation	tau phosphorylation, Aβ production, neuroinflammation<br><br>(*in vitro* and *in vivo*)	Cao, J., et al. (2021). Mol Psychiatry. 26 (9):4687-4701.
miR-196a	Downregulation	neuroinflammation, neuron apoptosis<br><br>(*in vivo*)	Yang, K., et al. (2019). J Cell Biochem. 120 (10):17811-17821.
miR-200	Downregulation	tau phosphorylation, neuron apoptosis<br><br>(*in vitro* and *in vivo*)	Park, H., et al. (2022). Int J Biol Sci. 18 (5):2220-2234.<br><br>Zhang, Q.S., et al. (2017). J Biosci. 42 (3):397-404.
miR-204-3p	Downregulation	Aβ deposition, microglia activation<br><br>(*in vitro* and *in vivo*)	Tao, W., et al. (2021). Mol Ther. 29 (1):396-408.
miR-206	Upregulation	Aβ burden, neurite growth, neuroinflammation<br><br>(*in vitro* and *in vivo*)	Peng, D., et al. (2022). Theranostics. 12 (8):3862-3881.<br><br>Xing, H., et al. (2016). Mol Med Rep. 14 (2):1357-1364.
miR-212	Downregulation	tau phosphorylation, Aβ deposition and production, neuroinflammation	Hernandez-Rapp, J., et al. (2016). Sci Rep. 6:30953.<br><br>Nong, W., et al. (2022). Bosn J Basic Med Sci. 22 (4):540-

		(*in vitro* and *in vivo*)	552.<br><br>Wang, Y., et al. (2017). Neurobiol Aging. 51:156-166.
miR-214	Downregulation	neuronal injury, neuroinflammation, neuron apoptosis<br><br>(*in vitro* and *in vivo*)	Hu, G., et al. (2022). Brain Res Bull. 178:164-172.<br><br>Zhou, Y., et al. (2021). Neuroscience. 455:52-64.
miR-216a-5p	Downregulation	Aβ deposition, neuroinflammation<br><br>(*in vivo*)	Shao, P. (2021). Brain Res. 1766:147511.
miR-218	Upregulation	tau phosphorylation<br><br>(*in vitro* and *in vivo*)	Xiong, Y.S., et al. (2015). Aging Cell. 14 (5):867-877.
miR-219-5p	Upregulation	tau phosphorylation<br><br>(*in vitro* and *in vivo*)	Li, J., et al. (2019). J Cell Biochem. 120 (6):9936-9946.
miR-224-5p	Downregulation	neuroinflammation<br><br>(*in vivo*)	Sun, X., et al. (2023). J Inflamm Res. 16:1639-1652.
miR-299-5p	Downregulation	neuron apoptosis<br><br>(*in vitro* and *in vivo*)	Zhang, Y., et al. (2016). Sci Rep. 6:24566.
miR-326	Downregulation	Aβ deposition, neuron apoptosis, tau phosphorylation<br><br>(*in vivo*)	He, B., et al. (2020). J Cell Physiol. 235 (1):480-493.
miR-335-5p	Downregulation	apoptosis, Aβ accumulation<br><br>(*in vitro* and *in vivo*)	Wang, D., et al. (2020). Curr Neurovasc Res. 17 (1):93-101.
miR-338-5p	Downregulation	Aβ formation and accumulation,	Qian, Q., et al. (2019). Faseb

		neuroinflammation (*in vivo*)	j. 33 (3):4404-4417.
miR-340	Downregulation	Aβ accumulation, neuronal apoptosis (*in vitro* and *in vivo*)	Tan, X., et al. (2020). Curr Neurovasc Res. 17 (1):86-92.
miR-342-3p	Upregulation	Aβ burden, neuronal apoptosis (*in vitro* and *in vivo*)	Fu, Y., et al. (2019). Metab Brain Dis. 34 (5):1355-1363.
miR-369	Downregulation	tau phosphorylation (*in vivo*)	Yao, X., et al. (2019). Front Aging Neurosci. 11:365.
miR-375	Upregulation	neuronal apoptosis (*in vitro* and *in vivo*)	Wang, Q., et al. (2020). Aging (Albany NY). 12 (23):23974-23995.
miR-425	Downregulation	Aβ production and aggregation, neuron damage (*in vitro* and *in vivo*)	Hu, Y.B., et al. (2021). Aging Cell. 20 (10):e13454.
miR-451a	Downregulation	neuroinflammation, Aβ load (*in vivo*)	Feng, H., et al. (2023). Theranostics. 13 (9):3021-3040.
miR-485-3p	Upregulation	Aβ accumulation, tau phosphorylation, neuroinflammation (*in vitro* and *in vivo*)	Koh, H.S., et al. (2021). Int J Mol Sci. 22 (23).
miR-504-3p	Downregulation	tau hyperphosphorylation (*in vitro* and *in vivo*)	Chen, D., et al. (2022). Transl Neurodegener. 11 (1):27.
miR-511-3p	Downregulation	neuroinflammation	Wang, T., et al. (2023). Exp

		(*in vitro* and *in vivo*)	Gerontol. 178:112195.
miR-539-5p	Downregulation	Aβ accumulation, tau phosphorylation, apoptosis<br><br>(*in vivo*)	Jiang, Y., et al. (2020). Neurotox Res. 38 (2):524-535.
miR-590-5p	Downregulation	cell apoptosis<br><br>(*in vitro* and *in vivo*)	Shang, L., et al. (2022). Anal Cell Pathol (Amst). 2022:7657995.
miR-937	Upregulation	Aβ deposition<br><br>(*in vivo*)	Liu, Z., et al. (2015). Cell Physiol Biochem. 37 (1):321-330.
miR-3142	remains to be further elucidated	cell apoptosis<br><br>(*in vitro* and *in vivo*)	Tang, Z.B., et al. (2022). Brain Res. 1785:147884.

2 References

Abozaid, O.A.R., Sallam, M.W., El-Sonbaty, S., Aziza, S., Emad, B., Ahmed, E.S.A. (2022). Resveratrol-Selenium Nanoparticles Alleviate Neuroinflammation and Neurotoxicity in a Rat Model of Alzheimer's Disease by Regulating Sirt1/miRNA-134/GSK3β Expression. Biol Trace Elem Res. 200 (12):5104-5114. doi: 10.1007/s12011-021-03073-7.

Banzhaf-Strathmann, J., Benito, E., May, S., Arzberger, T., Tahirovic, S., Kretzschmar, H., et al. (2014). MicroRNA-125b induces tau hyperphosphorylation and cognitive deficits in Alzheimer's disease. Embo j. 33 (15):1667-1680. doi: 10.15252/embj.201387576.

Barros-Viegas, A.T., Carmona, V., Ferreiro, E., Guedes, J., Cardoso, A.M., Cunha, P., et al. (2020). miRNA-31 Improves Cognition and Abolishes Amyloid-β Pathology by Targeting APP and BACE1 in an Animal Model of Alzheimer's Disease. Mol Ther Nucleic Acids. 19:1219-1236. doi: 10.1016/j.omtn.2020.01.010.

Boscher, E., Goupil, C., Petry, S., Keraudren, R., Loiselle, A., Planel, E., et al. (2020). MicroRNA-138 Overexpression Alters $A\beta_{42}$ Levels and Behavior in Wildtype Mice. Front Neurosci. 14:591138. doi: 10.3389/fnins.2020.591138.

Boscher, E., Husson, T., Quenez, O., Laquerrière, A., Marguet, F., Cassinari, K., et al. (2019). Copy Number Variants in miR-138 as a Potential Risk Factor for Early-Onset Alzheimer's Disease. J Alzheimers Dis. 68 (3):1243-1255. doi: 10.3233/jad-180940.

Cao, J., Huang, M., Guo, L., Zhu, L., Hou, J., Zhang, L., et al. (2021). MicroRNA-195 rescues ApoE4-induced cognitive deficits and lysosomal defects in Alzheimer's disease pathogenesis. Mol Psychiatry. 26 (9):4687-4701. doi: 10.1038/s41380-020-0824-3.

Chen, D., Lan, G., Li, R., Mei, Y., Shui, X., Gu, X., et al. (2022). Melatonin ameliorates tau-related pathology via the miR-504-3p and CDK5 axis in Alzheimer's disease. Transl Neurodegener. 11 (1):27. doi: 10.1186/s40035-022-00302-4.

Chu, Y.Y., Ko, C.Y., Wang, W.J., Wang, S.M., Gean, P.W., Kuo, Y.M., et al. (2016). Astrocytic CCAAT/Enhancer Binding Protein δ Regulates Neuronal Viability and Spatial Learning Ability via miR-135a. Mol Neurobiol. 53 (6):4173-4188. doi: 10.1007/s12035-015-9359-z.

Duan, Q., Si, E. (2019). MicroRNA-25 aggravates $A\beta_{1-42}$-induced hippocampal neuron injury in Alzheimer's disease by downregulating KLF2 via the Nrf2 signaling pathway in a mouse model. J Cell Biochem. 120 (9):15891-15905. doi: 10.1002/jcb.28861.

Feng, H., Hu, P., Chen, Y., Sun, H., Cai, J., He, X., et al. (2023). Decreased miR-451a in cerebrospinal fluid, a marker for both cognitive impairment and depressive symptoms in Alzheimer's disease. Theranostics. 13 (9):3021-3040. doi: 10.7150/thno.81826.

Fu, Y., Hu, X., Zheng, C., Sun, G., Xu, J., Luo, S., et al. (2019). Intrahippocampal miR-342-3p inhibition reduces β-amyloid plaques and ameliorates learning and memory in Alzheimer's disease. Metab Brain Dis. 34 (5):1355-1363. doi: 10.1007/s11011-019-00438-9.

Garcia, G., Fernandes, A., Stein, F., Brites, D. (2022). Protective Signature of IFNγ-Stimulated Microglia Relies on miR-124-3p Regulation From the Secretome Released by Mutant APP Swedish Neuronal Cells. Front Pharmacol. 13:833066. doi: 10.3389/fphar.2022.833066.

Guedes, J.R., Custódia, C.M., Silva, R.J., de Almeida, L.P., Pedroso de Lima, M.C., Cardoso, A.L. (2014). Early miR-155 upregulation contributes to neuroinflammation in Alzheimer's disease triple transgenic mouse model. Hum Mol Genet. 23 (23):6286-6301. doi: 10.1093/hmg/ddu348.

Han, C., Guo, L., Yang, Y., Guan, Q., Shen, H., Sheng, Y., et al. (2020). Mechanism of microRNA-22 in regulating neuroinflammation in Alzheimer's disease. Brain Behav. 10 (6):e01627. doi: 10.1002/brb3.1627.

He, B., Chen, W., Zeng, J., Tong, W., Zheng, P. (2020). MicroRNA-326 decreases tau phosphorylation and neuron apoptosis through inhibition of the JNK signaling pathway by targeting VAV1 in Alzheimer's disease. J Cell Physiol. 235 (1):480-493. doi: 10.1002/jcp.28988.

Hébert, S.S., Horré, K., Nicolaï, L., Papadopoulou, A.S., Mandemakers, W., Silahtaroglu, A.N., et al. (2008). Loss of microRNA cluster miR-29a/b-1 in sporadic Alzheimer's disease correlates with increased BACE1/beta-secretase expression. Proc Natl Acad Sci U S A. 105 (17):6415-6420. doi: 10.1073/pnas.0710263105.

Hernandez-Rapp, J., Rainone, S., Goupil, C., Dorval, V., Smith, P.Y., Saint-Pierre, M., et al. (2016). microRNA-132/212 deficiency enhances Aβ production and senile plaque deposition in Alzheimer's disease triple transgenic mice. Sci Rep. 6:30953. doi: 10.1038/srep30953.

Hou, T.Y., Zhou, Y., Zhu, L.S., Wang, X., Pang, P., Wang, D.Q., et al. (2020). Correcting abnormalities in miR-124/PTPN1 signaling rescues tau pathology in Alzheimer's disease. J Neurochem. 154 (4):441-457. doi: 10.1111/jnc.14961.

Hu, G., Shi, Z., Shao, W., Xu, B. (2022). MicroRNA-214-5p involves in the protection effect of Dexmedetomidine against neurological injury in Alzheimer's disease via targeting the suppressor of zest 12. Brain Res Bull. 178:164-172. doi: 10.1016/j.brainresbull.2021.10.016.

Hu, Y., Wu, L., Jiang, L., Liang, N., Zhu, X., He, Q., et al. (2021). Notoginsenoside R2 reduces $A\beta_{25-35}$-induced neuronal apoptosis and inflammation via miR-27a/SOX8/β-catenin axis. Hum Exp Toxicol. 40 (12_suppl):S347-s358. doi: 10.1177/09603271211041996.

Hu, Y.B., Zhang, Y.F., Ren, R.J., Dammer, E.B., Xie, X.Y., Chen, S.W., et al. (2021). microRNA-425 loss mediates amyloid plaque microenvironment heterogeneity and promotes neurodegenerative pathologies. Aging Cell. 20 (10):e13454. doi: 10.1111/acel.13454.

Hu, Y.K., Wang, X., Li, L., Du, Y.H., Ye, H.T., Li, C.Y. (2013). MicroRNA-98 induces an Alzheimer's disease-like disturbance by targeting insulin-like growth factor 1. Neurosci Bull. 29 (6):745-751. doi: 10.1007/s12264-013-1348-5.

Jiang, H., Liu, J., Guo, S., Zeng, L., Cai, Z., Zhang, J., et al. (2022). miR-23b-3p rescues cognition in Alzheimer's disease by reducing tau phosphorylation and apoptosis via GSK-3β signaling pathways. Mol Ther Nucleic Acids. 28:539-557. doi: 10.1016/j.omtn.2022.04.008.

Jiang, Y., Xu, B., Chen, J., Sui, Y., Ren, L., Li, J., et al. (2018). Micro-RNA-137 Inhibits Tau Hyperphosphorylation in Alzheimer's Disease and Targets the CACNA1C Gene in Transgenic Mice and Human Neuroblastoma SH-SY5Y Cells. Med Sci Monit. 24:5635-5644. doi: 10.12659/msm.908765.

Jiang, Y., Zhang, Y., Su, L. (2020). MiR-539-5p Decreases amyloid β-protein production, hyperphosphorylation of Tau and Memory Impairment by Regulating PI3K/Akt/GSK-3β Pathways in APP/PS1 Double Transgenic Mice. Neurotox Res. 38 (2):524-535. doi: 10.1007/s12640-020-00217-w.

Jiao, Y., Kong, L., Yao, Y., Li, S., Tao, Z., Yan, Y., et al. (2016). Osthole decreases beta amyloid levels through up-regulation of miR-107 in Alzheimer's disease. Neuropharmacology. 108:332-344. doi: 10.1016/j.neuropharm.2016.04.046.

Khodabakhsh, P., Bazrgar, M., Mohagheghi, F., Parvardeh, S., Ahmadiani, A. (2023). MicroRNA-140-5p inhibitor attenuates memory impairment induced by amyloid-β oligomer in vivo possibly through Pin1 regulation. CNS Neurosci Ther. 29 (1):91-103. doi: 10.1111/cns.13980.

Kim, J., Yoon, H., Horie, T., Burchett, J.M., Restivo, J.L., Rotllan, N., et al. (2015). microRNA-33 Regulates ApoE Lipidation and Amyloid-β Metabolism in the Brain. J Neurosci. 35 (44):14717-14726. doi: 10.1523/jneurosci.2053-15.2015.

Kim, Y.J., Kim, S.H., Park, Y., Park, J., Lee, J.H., Kim, B.C., et al. (2020). miR-16-5p is upregulated by amyloid β deposition in Alzheimer's disease models and induces neuronal cell apoptosis through

direct targeting and suppression of BCL-2. Exp Gerontol. 136:110954. doi: 10.1016/j.exger.2020.110954.

Koh, H.S., Lee, S., Lee, H.J., Min, J.W., Iwatsubo, T., Teunissen, C.E., et al. (2021). Targeting MicroRNA-485-3p Blocks Alzheimer’s Disease Progression. Int J Mol Sci. 22 (23). doi: 10.3390/ijms222313136.

Li, J., Chen, W., Yi, Y., Tong, Q. (2019). miR-219-5p inhibits tau phosphorylation by targeting TTBK1 and GSK-3β in Alzheimer’s disease. J Cell Biochem. 120 (6):9936-9946. doi: 10.1002/jcb.28276.

Li, P., Xu, Y., Wang, B., Huang, J., Li, Q. (2020). miR-34a-5p and miR-125b-5p attenuate Aβ-induced neurotoxicity through targeting BACE1. J Neurol Sci. 413:116793. doi: 10.1016/j.jns.2020.116793.

Li, S., Poon, C.H., Zhang, Z., Yue, M., Chen, R., Zhang, Y., et al. (2023). MicroRNA-128 suppresses tau phosphorylation and reduces amyloid-beta accumulation by inhibiting the expression of GSK3β, APPBP2, and mTOR in Alzheimer’s disease. CNS Neurosci Ther. doi: 10.1111/cns.14143.

Li, Z., Chen, Q., Liu, J., Du, Y. (2020). Physical Exercise Ameliorates the Cognitive Function and Attenuates the Neuroinflammation of Alzheimer’s Disease via miR-129-5p. Dement Geriatr Cogn Disord. 49 (2):163-169. doi: 10.1159/000507285.

Liang, C., Mu, Y., Tian, H., Wang, D., Zhang, S., Wang, H., et al. (2021). MicroRNA-140 silencing represses the incidence of Alzheimer’s disease. Neurosci Lett. 758:135674. doi: 10.1016/j.neulet.2021.135674.

Liang, W., Xie, Z., Liao, D., Li, Y., Li, Z., Zhao, Y., et al. (2023). Inhibiting microRNA-142-5p improves learning and memory in Alzheimer’s disease rats via targeted regulation of the PTPN1-mediated Akt pathway. Brain Res Bull. 192:107-114. doi: 10.1016/j.brainresbull.2022.02.016.

Liu, D., Tang, H., Li, X.Y., Deng, M.F., Wei, N., Wang, X., et al. (2017). Targeting the HDAC2/HNF-4A/miR-101b/AMPK Pathway Rescues Tauopathy and Dendritic Abnormalities in Alzheimer’s Disease. Mol Ther. 25 (3):752-764. doi: 10.1016/j.ymthe.2017.01.018.

Liu, D.Y., Zhang, L. (2019). MicroRNA-132 promotes neurons cell apoptosis and activates Tau phosphorylation by targeting GTDC-1 in Alzheimer’s disease. Eur Rev Med Pharmacol Sci. 23 (19):8523-8532. doi: 10.26355/eurrev_201910_19166.

Liu, W., Zhao, J., Lu, G. (2016). miR-106b inhibits tau phosphorylation at Tyr18 by targeting Fyn in a model of Alzheimer’s disease. Biochem Biophys Res Commun. 478 (2):852-857. doi: 10.1016/j.bbrc.2016.08.037.

Liu, Y., Wang, L., Xie, F., Wang, X., Hou, Y., Wang, X., et al. (2020). Overexpression of miR-26a-5p Suppresses Tau Phosphorylation and Aβ Accumulation in the Alzheimer’s Disease Mice by Targeting DYRK1A. Curr Neurovasc Res. 17 (3):241-248. doi: 10.2174/1567202617666200414142637.

Liu, Y., Zhang, Y., Liu, P., Bai, H., Li, X., Xiao, J., et al. (2019). MicroRNA-128 knockout inhibits the development of Alzheimer's disease by targeting PPARγ in mouse models. Eur J Pharmacol. 843:134-144. doi: 10.1016/j.ejphar.2018.11.004.

Liu, Z., Wang, C., Wang, X., Xu, S. (2015). Therapeutic Effects of Transplantation of As-MiR-937-Expressing Mesenchymal Stem Cells in Murine Model of Alzheimer's Disease. Cell Physiol Biochem. 37 (1):321-330. doi: 10.1159/000430356.

Ma, P., Li, Y., Zhang, W., Fang, F., Sun, J., Liu, M., et al. (2019). Long Non-coding RNA MALAT1 Inhibits Neuron Apoptosis and Neuroinflammation While Stimulates Neurite Outgrowth and Its Correlation With MiR-125b Mediates PTGS2, CDK5 and FOXQ1 in Alzheimer's Disease. Curr Alzheimer Res. 16 (7):596-612. doi: 10.2174/1567205016666190725130134.

Mai, H., Fan, W., Wang, Y., Cai, Y., Li, X., Chen, F., et al. (2019). Intranasal Administration of miR-146a Agomir Rescued the Pathological Process and Cognitive Impairment in an AD Mouse Model. Mol Ther Nucleic Acids. 18:681-695. doi: 10.1016/j.omtn.2019.10.002.

Nong, W., Bao, C., Chen, Y., Wei, Z. (2022). miR-212-3p attenuates neuroinflammation of rats with Alzheimer's disease via regulating the SP1/BACE1/NLRP3/Caspase-1 signaling pathway. Bosn J Basic Med Sci. 22 (4):540-552. doi: 10.17305/bjbms.2021.6723.

Pan, K., Chen, S., Wang, Y., Yao, W., Gao, X. (2021). MicroRNA-23b attenuates tau pathology and inhibits oxidative stress by targeting GnT-III in Alzheimer's disease. Neuropharmacology. 196:108671. doi: 10.1016/j.neuropharm.2021.108671.

Park, H., Lee, Y.B., Chang, K.A. (2022). miR-200c suppression increases tau hyperphosphorylation by targeting 14-3-3γ in early stage of 5×FAD mouse model of Alzheimer's disease. Int J Biol Sci. 18 (5):2220-2234. doi: 10.7150/ijbs.66604.

Parsi, S., Smith, P.Y., Goupil, C., Dorval, V., Hébert, S.S. (2015). Preclinical Evaluation of miR-15/107 Family Members as Multifactorial Drug Targets for Alzheimer's Disease. Mol Ther Nucleic Acids. 4 (10):e256. doi: 10.1038/mtna.2015.33.

Peng, D., Wang, Y., Xiao, Y., Peng, M., Mai, W., Hu, B., et al. (2022). Extracellular vesicles derived from astrocyte-treated with haFGF(14-154) attenuate Alzheimer phenotype in AD mice. Theranostics. 12 (8):3862-3881. doi: 10.7150/thno.70951.

Qian, Q., Zhang, J., He, F.P., Bao, W.X., Zheng, T.T., Zhou, D.M., et al. (2019). Down-regulated expression of microRNA-338-5p contributes to neuropathology in Alzheimer's disease. Faseb j. 33 (3):4404-4417. doi: 10.1096/fj.201801846R.

Ruan, Z., Li, Y., He, R., Li, X. (2021). Inhibition of microRNA-10b-5p up-regulates HOXD10 to attenuate Alzheimer's disease in rats via the Rho/ROCK signalling pathway. J Drug Target. 29 (5):531-540. doi: 10.1080/1061186x.2020.1864739.

Salta, E., Sierksma, A., Vanden Eynden, E., De Strooper, B. (2016). miR-132 loss de-represses ITPKB and aggravates amyloid and TAU pathology in Alzheimer's brain. EMBO Mol Med. 8 (9):1005-1018. doi: 10.15252/emmm.201606520.

Sarkar, S., Engler-Chiurazzi, E.B., Cavendish, J.Z., Povroznik, J.M., Russell, A.E., Quintana, D.D., et al. (2019). Over-expression of miR-34a induces rapid cognitive impairment and Alzheimer's disease-like pathology. Brain Res. 1721:146327. doi: 10.1016/j.brainres.2019.146327.

Shang, L., Peng, T., Chen, X., Yan, Z., Wang, J., Gao, X., et al. (2022). miR-590-5p Overexpression Alleviates β-Amyloid-Induced Neuron Damage via Targeting Pellino-1. Anal Cell Pathol (Amst). 2022:7657995. doi: 10.1155/2022/7657995.

Shao, P. (2021). MiR-216a-5p ameliorates learning-memory deficits and neuroinflammatory response of Alzheimer's disease mice via regulation of HMGB1/NF-κB signaling. Brain Res. 1766:147511. doi: 10.1016/j.brainres.2021.147511.

Shu, B., Zhang, X., Du, G., Fu, Q., Huang, L. (2018). MicroRNA-107 prevents amyloid-β-induced neurotoxicity and memory impairment in mice. Int J Mol Med. 41 (3):1665-1672. doi: 10.3892/ijmm.2017.3339.

Song, C., Shi, J., Xu, J., Zhao, L., Zhang, Y., Huang, W., et al. (2021). Post-transcriptional regulation of α7 nAChR expression by miR-98-5p modulates cognition and neuroinflammation in an animal model of Alzheimer's disease. Faseb j. 35 (6):e21658. doi: 10.1096/fj.202100257R.

Sun, T., Zhao, K., Liu, M., Cai, Z., Zeng, L., Zhang, J., et al. (2022). miR-30a-5p induces Aβ production via inhibiting the nonamyloidogenic pathway in Alzheimer's disease. Pharmacol Res. 178:106153. doi: 10.1016/j.phrs.2022.106153.

Sun, W., Zhao, J., Li, C. (2020). Dexmedetomidine Provides Protection Against Hippocampal Neuron Apoptosis and Cognitive Impairment in Mice with Alzheimer's Disease by Mediating the miR-129/YAP1/JAG1 Axis. Mol Neurobiol. 57 (12):5044-5055. doi: 10.1007/s12035-020-02069-z.

Sun, X., Deng, Y., Ge, P., Peng, Q., Soufiany, I., Zhu, L., et al. (2023). Diminazene Ameliorates Neuroinflammation by Suppression of Astrocytic miRNA-224-5p/NLRP3 Axis in Alzheimer's Disease Model. J Inflamm Res. 16:1639-1652. doi: 10.2147/jir.S401385.

Tan, X., Luo, Y., Pi, D., Xia, L., Li, Z., Tu, Q. (2020). MiR-340 Reduces the Accumulation of Amyloid-β Through Targeting BACE1 (β-site Amyloid Precursor Protein Cleaving Enzyme 1) in Alzheimer's Disease. Curr Neurovasc Res. 17 (1):86-92. doi: 10.2174/1567202617666200117103931.

Tang, Y., Bao, J.S., Su, J.H., Huang, W. (2017). MicroRNA-139 modulates Alzheimer's-associated pathogenesis in SAMP8 mice by targeting cannabinoid receptor type 2. Genet Mol Res. 16 (1). doi: 10.4238/gmr16019166.

Tang, Z.B., Chen, H.P., Zhong, D., Song, J.H., Cao, J.W., Zhao, M.Q., et al. (2022). LncRNA RMRP accelerates autophagy-mediated neurons apoptosis through miR-3142/TRIB3 signaling axis in alzheimer's disease. Brain Res. 1785:147884. doi: 10.1016/j.brainres.2022.147884.

Tao, W., Yu, L., Shu, S., Liu, Y., Zhuang, Z., Xu, S., et al. (2021). miR-204-3p/Nox4 Mediates Memory Deficits in a Mouse Model of Alzheimer's Disease. Mol Ther. 29 (1):396-408. doi: 10.1016/j.ymthe.2020.09.006.

Vilardo, E., Barbato, C., Ciotti, M., Cogoni, C., Ruberti, F. (2010). MicroRNA-101 regulates amyloid precursor protein expression in hippocampal neurons. J Biol Chem. 285 (24):18344-18351. doi: 10.1074/jbc.M110.112664.

Wang, D., Fei, Z., Luo, S., Wang, H. (2020). MiR-335-5p Inhibits β-Amyloid (Aβ) Accumulation to Attenuate Cognitive Deficits Through Targeting c-jun-N-terminal Kinase 3 in Alzheimer's Disease. Curr Neurovasc Res. 17 (1):93-101. doi: 10.2174/1567202617666200128141938.

Wang, G., Huang, Y., Wang, L.L., Zhang, Y.F., Xu, J., Zhou, Y., et al. (2016). MicroRNA-146a suppresses ROCK1 allowing hyperphosphorylation of tau in Alzheimer's disease. Sci Rep. 6:26697. doi: 10.1038/srep26697.

Wang, L., Shui, X., Mei, Y., Xia, Y., Lan, G., Hu, L., et al. (2022). miR-143-3p Inhibits Aberrant Tau Phosphorylation and Amyloidogenic Processing of APP by Directly Targeting DAPK1 in Alzheimer's Disease. Int J Mol Sci. 23 (14). doi: 10.3390/ijms23147992.

Wang, L., Shui, X., Zhang, M., Mei, Y., Xia, Y., Lan, G., et al. (2022). MiR-191-5p Attenuates Tau Phosphorylation, Aβ Generation, and Neuronal Cell Death by Regulating Death-Associated Protein Kinase 1. ACS Chem Neurosci. 13 (24):3554-3566. doi: 10.1021/acschemneuro.2c00423.

Wang, Q., Ge, X., Zhang, J., Chen, L. (2020). Effect of lncRNA WT1-AS regulating WT1 on oxidative stress injury and apoptosis of neurons in Alzheimer's disease via inhibition of the miR-375/SIX4 axis. Aging (Albany NY). 12 (23):23974-23995. doi: 10.18632/aging.104079.

Wang, T., Long, Q., Hu, Y., Yang, Y., Li, X., Wei, H. (2023). miR-181c-5p suppresses neuronal pyroptosis via NLRP1 in Alzheimer's disease. Behav Brain Res. 447:114387. doi: 10.1016/j.bbr.2023.114387.

Wang, T., Zhao, W., Liu, Y., Yang, D., He, G., Wang, Z. (2023). MicroRNA-511-3p regulates $A\beta_{1-40}$ induced decreased cell viability and serves as a candidate biomarker in Alzheimer's disease. Exp Gerontol. 178:112195. doi: 10.1016/j.exger.2023.112195.

Wang, W., Gu, X.H., Li, M., Cheng, Z.J., Tian, S., Liao, Y., et al. (2022). MicroRNA-155-5p Targets SKP2, Activates IKKβ, Increases Aβ Aggregation, and Aggravates a Mouse Alzheimer Disease Model. J Neuropathol Exp Neurol. 81 (1):16-26. doi: 10.1093/jnen/nlab116.

Wang, Y., Veremeyko, T., Wong, A.H., El Fatimy, R., Wei, Z., Cai, W., et al. (2017). Downregulation of miR-132/212 impairs S-nitrosylation balance and induces tau phosphorylation in Alzheimer's disease. Neurobiol Aging. 51:156-166. doi: 10.1016/j.neurobiolaging.2016.12.015.

Wu, Q., Yuan, X., Bai, J., Han, R., Li, Z., Zhang, H., et al. (2019). MicroRNA-181a protects against pericyte apoptosis via directly targeting FOXO1: implication for ameliorated cognitive deficits in APP/PS1 mice. Aging (Albany NY). 11 (16):6120-6133. doi: 10.18632/aging.102171.

Xing, H., Guo, S., Zhang, Y., Zheng, Z., Wang, H. (2016). Upregulation of microRNA-206 enhances lipopolysaccharide-induced inflammation and release of amyloid-β by targeting insulin-like growth factor 1 in microglia. Mol Med Rep. 14 (2):1357-1364. doi: 10.3892/mmr.2016.5369.

Xiong, Y.S., Liu, F.F., Liu, D., Huang, H.Z., Wei, N., Tan, L., et al. (2015). Opposite effects of two estrogen receptors on tau phosphorylation through disparate effects on the miR-218/PTPA pathway. Aging Cell. 14 (5):867-877. doi: 10.1111/acel.12366.

Yan, Y., Yan, H., Teng, Y., Wang, Q., Yang, P., Zhang, L., et al. (2020). Long non-coding RNA 00507/miRNA-181c-5p/TTBK1/MAPT axis regulates tau hyperphosphorylation in Alzheimer's disease. J Gene Med. 22 (12):e3268. doi: 10.1002/jgm.3268.

Yang, K., Feng, S., Ren, J., Zhou, W. (2019). Upregulation of microRNA-196a improves cognitive impairment and alleviates neuronal damage in hippocampus tissues of Alzheimer's disease through downregulating LRIG3 expression. J Cell Biochem. 120 (10):17811-17821. doi: 10.1002/jcb.29047.

Yao, X., Xian, X., Fang, M., Fan, S., Li, W. (2019). Loss of miR-369 Promotes Tau Phosphorylation by Targeting the Fyn and Serine/Threonine-Protein Kinase 2 Signaling Pathways in Alzheimer's Disease Mice. Front Aging Neurosci. 11:365. doi: 10.3389/fnagi.2019.00365.

Ye, X., Luo, H., Chen, Y., Wu, Q., Xiong, Y., Zhu, J., et al. (2015). MicroRNAs 99b-5p/100-5p Regulated by Endoplasmic Reticulum Stress are Involved in Abeta-Induced Pathologies. Front Aging Neurosci. 7:210. doi: 10.3389/fnagi.2015.00210.

Zeng, L., Jiang, H., Ashraf, G.M., Liu, J., Wang, L., Zhao, K., et al. (2021). Implications of miR-148a-3p/p35/PTEN signaling in tau hyperphosphorylation and autoregulatory feedforward of Akt/CREB in Alzheimer's disease. Mol Ther Nucleic Acids. 27:256-275. doi: 10.1016/j.omtn.2021.11.019.

Zhai, L., Shen, H., Sheng, Y., Guan, Q. (2021). ADMSC Exo-MicroRNA-22 improve neurological function and neuroinflammation in mice with Alzheimer's disease. J Cell Mol Med. 25 (15):7513-7523. doi: 10.1111/jcmm.16787.

Zhang, J., Hu, M., Teng, Z., Tang, Y.P., Chen, C. (2014). Synaptic and cognitive improvements by inhibition of 2-AG metabolism are through upregulation of microRNA-188-3p in a mouse model of Alzheimer's disease. J Neurosci. 34 (45):14919-14933. doi: 10.1523/jneurosci.1165-14.2014.

Zhang, Q.S., Liu, W., Lu, G.X. (2017). miR-200a-3p promotes β-Amyloid-induced neuronal apoptosis through down-regulation of SIRT1 in Alzheimer's disease. J Biosci. 42 (3):397-404. doi: 10.1007/s12038-017-9698-1.

Zhang, Y., Liu, C., Wang, J., Li, Q., Ping, H., Gao, S., et al. (2016). MiR-299-5p regulates apoptosis through autophagy in neurons and ameliorates cognitive capacity in APPswe/PS1dE9 mice. Sci Rep. 6:24566. doi: 10.1038/srep24566.

Zheng, K., Hu, F., Zhou, Y., Zhang, J., Zheng, J., Lai, C., et al. (2021). miR-135a-5p mediates memory and synaptic impairments via the Rock2/Adducin1 signaling pathway in a mouse model of Alzheimer's disease. Nat Commun. 12 (1):1903. doi: 10.1038/s41467-021-22196-y.

Zhou, Y., Deng, J., Chu, X., Zhao, Y., Guo, Y. (2019). Role of Post-Transcriptional Control of Calpain by miR-124-3p in the Development of Alzheimer's Disease. J Alzheimers Dis. 67 (2):571-581. doi: 10.3233/jad-181053.

Zhou, Y., Ge, Y., Liu, Q., Li, Y.X., Chao, X., Guan, J.J., et al. (2021). LncRNA BACE1-AS Promotes Autophagy-Mediated Neuronal Damage Through The miR-214-3p/ATG5 Signalling Axis In Alzheimer's Disease. Neuroscience. 455:52-64. doi: 10.1016/j.neuroscience.2020.10.028.

Zhu, M., Jia, L., Jia, J. (2021). Inhibition of miR-96-5p May Reduce $A\beta_{42}/A\beta_{40}$ Ratio via Regulating ATP-binding cassette transporter A1. J Alzheimers Dis. 83 (1):367-377. doi: 10.3233/jad-210411.
